# Fruits, Vegetables, and Legumes: Harnessing the Gut-Kidney Axis in Hypertensive Chronic Kidney Disease

**DOI:** 10.3390/foods15162938

**Published:** 2026-08-21

**Authors:** Maya Agustin, Edwin Hadinata, Dante Saksono Harbuwono, Hanselian Trixie, Antonello Santini, Fahrul Nurkolis

**Affiliations:** 1 Fahrul Institute for Innovation and Research (FIIR), Yogyakarta 55281, Indonesiafahrul.nurkolis.mail@gmail.com (F.N.); 2 Faculty of Medicine, Universitas Ciputra Surabaya, Surabaya 60219, Indonesia; 3 Division of Endocrinology, Metabolism, and Diabetes, Department of Internal Medicine, Faculty of Medicine, Universitas Indonesia, Dr. Cipto Mangunkusumo National Referral Hospital, Jakarta 10430, Indonesia; 4 Department of Pharmacy, University of Napoli Federico II, Via Domenico Montesano, 49, 80131 Napoli, Italy; 5 Medical Research Center of Indonesia, Surabaya 60281, Indonesia; 6 Institute for Research and Community Service, State Islamic University of Sunan Kalijaga (UIN Sunan Kalijaga), Yogyakarta 55281, Indonesia; 7 Faculty of Medicine, Universitas Airlangga, Surabaya 60131, Indonesia

**Keywords:** chronic kidney disease, hypertension, gut microbiota, hyperkalemia, dietary potassium, fruits, vegetables, legumes, plant-based diet, short-chain fatty acids, uremic toxins

## Abstract

Hypertension is the most common comorbidity in chronic kidney disease (CKD), affecting roughly 80–85% of patients overall and an even higher proportion in nondialysis CKD G3–G5. This narrative review examines whether, and under what clinical conditions, fruits, vegetables, and legumes can be used safely in adults with hypertensive CKD, especially nondialysis CKD G3–G5, as well as moderate to severe loss of kidney function, paying close attention to the gut microbiota, short-chain fatty acids, gut-derived uremic toxins, potassium bioavailability, and modifiable clinical risk factors. The clinical effect of plant foods on serum potassium cannot be predicted from total potassium content alone. The Kidney Disease: Improving Global Outcomes (KDIGO) 2024 guidelines explicitly advise individualized potassium management and emphasize limiting foods rich in bioavailable potassium, especially processed foods, rather than reflexively restricting fruits and vegetables. Across CKD studies, the association between dietary potassium and serum potassium is weak or inconsistent, and the risk of hyperkalemia is modified by kidney function, bowel function, metabolic acidosis, diabetes and hyperglycemia, medications, food processing, and the food matrix itself. Potassium bioavailability varies substantially according to the food matrix, preparation method, portion size, and processing method; some minimally processed plant foods may provide less readily absorbable potassium than potassium-rich processed foods, juices, or potassium additives. Fruits, vegetables, and legumes also provide fiber, resistant starch, and polyphenols that can support saccharolytic fermentation, short-chain fatty acid production, bowel transit, and lower concentrations of some gut-derived uremic toxins. However, blood-pressure and kidney-outcome benefits in CKD are supported more strongly by observational and selected alkali-diet trials than by large microbiome-focused randomized studies. In hypertensive CKD, fruits, vegetables, and legumes should not be universally restricted simply because they contain potassium. A safer and more evidence-aligned strategy is individualized, food-source-aware prescribing that prioritizes minimally processed plant foods, accounts for preparation method and portion size, corrects constipation and acidosis, reviews potassium-raising medications and additives, and monitors serum potassium according to baseline risk.

## 1. Introduction

Hypertension is central to the clinical burden of chronic kidney disease (CKD) [1,2]. Contemporary nephrology guidance notes that hypertension affects about 80–85% of people with CKD and becomes even more prevalent as kidney function declines, reaching about 95% in CKD G4–G5 before kidney replacement therapy [3]. Elevated blood pressure accelerates albuminuria, cardiovascular complications, and kidney function loss, which is why Kidney Disease, Improving Global Outcomes (KDIGO) continues to emphasize strict blood-pressure control and sustained renin–angiotensin system inhibition when tolerated. At the same time, a treatment model focused only on sodium restriction and antihypertensive drugs is incomplete because it does not address dietary quality, acid load, bowel function, microbiota metabolism, or the nutritional consequences of overly restrictive diets [3].

Hypertension in CKD arises from converging abnormalities in volume and vascular regulation. Reduced renal sodium excretion promotes salt and volume expansion, while sympathetic nervous system overactivity and activation of the renin–angiotensin–aldosterone system further increase vascular resistance, sodium retention, and blood pressure. Endothelial dysfunction, oxidative stress, and vascular remodeling contribute to arterial stiffening and impaired vasodilatory capacity, while secondary hyperaldosteronism may further promote sodium retention and vascular dysfunction. These mechanisms are frequently amplified by comorbid conditions common in CKD, including diabetes, obesity, obstructive sleep apnea, and anemia, which can worsen sympathetic activity, vascular dysfunction, volume status, or treatment resistance. Thus, hypertension in CKD represents a multifactorial phenotype in which renal, neurohormonal, vascular, and metabolic abnormalities interact rather than a consequence of impaired renal sodium handling alone [4,5].

The gut-kidney axis has become clinically important because CKD alters the intestinal environment in ways that favor dysbiosis and accumulation of harmful microbial metabolites [6]. Reviews of human and translational data describe increased urea diffusion into the gut lumen, disruption of intestinal epithelial integrity, greater permeability, altered motility, and a shift toward a proinflammatory intestinal milieu. These changes can foster endotoxemia, systemic inflammation, vascular dysfunction, and retention of gut-derived toxins such as indoxyl sulfate and p-cresyl sulfate [6].

This creates an important clinical tension in CKD dietary management. Fruits, vegetables, and legumes supply fermentable fiber, resistant starch, polyphenols, and plant protein, dietary features that may improve bowel transit, microbial ecology, metabolic acidosis, blood pressure, and diet quality [7,8,9]. Yet these same foods are often restricted because of their potassium content [10,11]. Low-potassium dietary practices can unintentionally reduce fiber intake, worsen constipation, narrow food variety, and increase reliance on ultra-processed or low-fiber foods; qualitative work in CKD also suggests that potassium restrictions are burdensome and may reduce quality of life [12,13,14].

**Figure 1:**
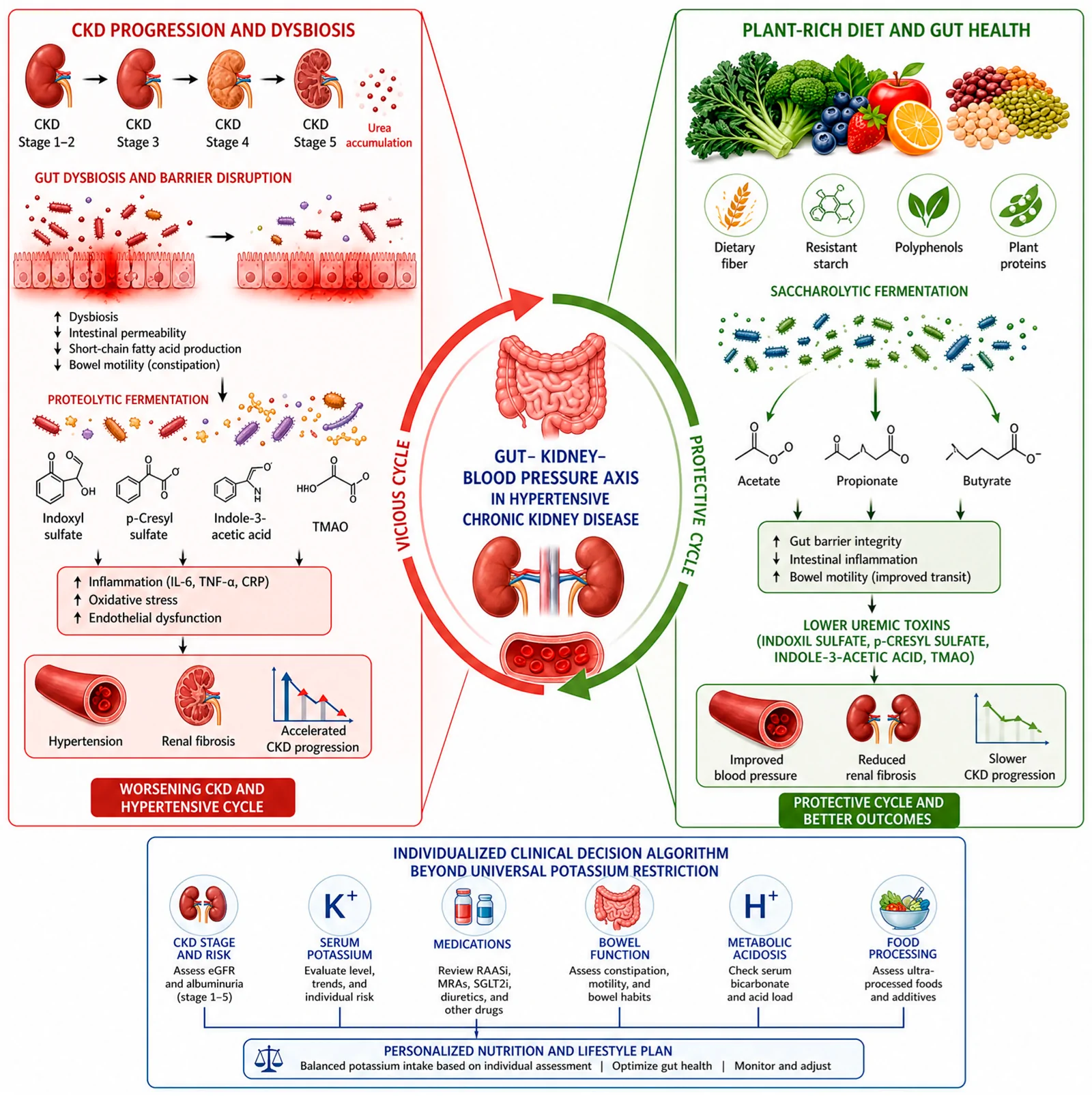
The Gut-Kidney-Blood Pressure Axis and the Central Role of Plant Foods in Hypertensive Chronic Kidney Disease. Overview of the gut-kidney-blood pressure axis in hypertensive chronic kidney disease and the proposed mechanisms through which fruits, vegetables, and legumes may influence intestinal homeostasis, gut-derived uremic metabolites, blood pressure regulation, and CKD-related outcomes, while emphasizing individualized potassium management.

The principal population considered in this review is adults with nondialysis CKD G3–G5, with particular attention to patients with hypertension. Dialysis populations are discussed only where they clarify mechanism or management. Mechanistic evidence is separated, as far as possible, from evidence strong enough to support clinical recommendations. The overall conceptual framework of this review, illustrating how CKD-associated dysbiosis, plant-food-derived microbial metabolism, and individualized potassium management interact within the gut-kidney-blood pressure axis, is presented in Figure 1.

Throughout this review, CKD severity is described using the KDIGO GFR-category nomenclature (G1–G5), with G3 further divided into G3a and G3b. The principal population of interest is adults with nondialysis CKD G3–G5, particularly those with G3b–G4 and hypertension. CKD G5 denotes kidney failure (eGFR < 15 mL/min/1.73 m^2^) but does not by itself imply dialysis dependence; therefore, dialysis-dependent populations are explicitly identified as such when discussed. Studies involving dialysis populations are included only when they provide relevant mechanistic or management evidence, and their findings are not assumed to be directly generalizable to the principal nondialysis population.

The purpose of this narrative review is to critically evaluate how fruits, vegetables, and legumes can be incorporated into dietary management of hypertensive CKD without relying on potassium content alone as the determinant of dietary restriction. Specifically, we integrate evidence across four interconnected domains, gut dysbiosis, saccharolytic and proteolytic fermentation, and gut-derived uremic toxins; the potential effects of fiber, resistant starch, polyphenols, and plant protein on the gut-kidney-blood pressure axis; potassium bioavailability as influenced by food matrix, processing, preparation, and portion size; and clinical and non-dietary modifiers of hyperkalemia risk, including kidney function, metabolic acidosis, constipation, diabetes, and potassium-raising medications. Unlike reviews that primarily examine plant-based diets, dietary potassium restriction, or the gut-kidney axis separately, this review brings these domains together within a risk-stratified framework for hypertensive nondialysis CKD. A central gap in the current literature is the limited integration of microbiome-related mechanisms with the practical problem of potassium safety. This review therefore aims to distinguish mechanistic evidence from clinical evidence, identify where evidence is observational or intervention-based, and translate these findings into a practical framework for individualized plant-food selection and potassium-risk management.

## 2. Methods

A narrative literature review was conducted to synthesize current evidence regarding the role of fruits, vegetables, and legumes in modulating the gut-kidney axis among adults with hypertensive chronic kidney disease (CKD). A structured literature search was performed in PubMed/MEDLINE, Scopus, Web of Science, and the Cochrane Library to identify relevant publications available up to July 2026. Search terms combined controlled vocabulary and free-text keywords related to chronic kidney disease, hypertension, gut microbiota, gut-kidney axis, fruits, vegetables, legumes, dietary fiber, resistant starch, polyphenols, potassium, hyperkalemia, short-chain fatty acids (SCFAs), and uremic toxins. Boolean operators (“AND” and “OR”) were used to combine search terms, and reference lists of eligible articles and relevant reviews were manually screened to identify additional pertinent studies.

Titles, abstracts, and potentially eligible full texts were assessed by the authors for relevance, with uncertainties discussed collectively and resolved through consensus. The review prioritized clinical practice guidelines, systematic reviews and meta-analyses, randomized controlled trials, prospective cohort studies, and mechanistic human studies addressing dietary plant foods, potassium management, gut microbiota, and CKD outcomes. Animal and in vitro studies were included only when they provided essential mechanistic insights not yet established in humans. Studies focusing exclusively on dialysis populations were considered only when they contributed to understanding biological mechanisms or informed clinical management relevant to nondialysis CKD. Literature selection emphasized methodological quality, clinical relevance, and recency of evidence, while recognizing the inherent limitations of a narrative review, including the absence of formal risk-of-bias assessment or quantitative evidence synthesis. Evidence was critically interpreted with particular attention to distinguishing mechanistic findings from data sufficient to support clinical recommendations. Because this was a narrative review, no formal risk-of-bias assessment or quantitative synthesis was undertaken.

To make the strength of evidence explicit throughout this review, findings are labeled using four descriptors. Established clinical evidence denotes conclusions supported by clinical practice guidelines (e.g., KDIGO 2024), randomized controlled trials (RCTs), or meta-analyses of trials conducted in human CKD populations. Observational (associational) evidence denotes prospective or cross-sectional cohort data that describe associations but cannot, on their own, establish causation. Mechanistic or preclinical evidence denotes in vitro, animal, or translational human studies that support biological plausibility but have not been confirmed as clinical benefit. Proposed mechanism or hypothesis denotes inferences that remain to be tested directly in CKD. Where a statement combines several tiers, the highest applicable tier and its principal limitation are indicated. This labeling is applied with particular care to claims regarding potassium homeostasis, inflammation, gut-derived uremic toxins, and kidney outcomes, for which biological plausibility and confirmed clinical benefit frequently diverge.

## 3. The Gut-Kidney-Blood Pressure Axis

CKD changes the intestinal environment in several directions at once. Greater delivery of urea to the gut, altered luminal chemistry, slower transit, low-fiber intake, medication effects, and epithelial injury together promote dysbiosis and barrier dysfunction [15,16]. Constipation is especially relevant because it is common in CKD, is driven by low dietary fiber, reduced activity, fluid restriction in some patients, and medication use, and is increasingly linked not only to symptom burden but also to renal and cardiovascular risk [16,17].

A useful way to frame CKD dysbiosis is the balance between saccharolytic fermentation and proteolytic fermentation [18,19,20,21]. When fermentable carbohydrates are available, the microbiota produce short-chain fatty acids such as acetate, propionate, and butyrate [22,23]. When fiber intake is low and intestinal transit is slow, colonic metabolism shifts toward proteolytic fermentation of amino acids, which increases generation of indole-, p-cresol-, and phenyl-derived toxins [20,24,25]. Observational work in CKD shows that dietary patterns with a higher plant-fiber-to-protein balance are associated with lower uremic toxin concentrations, supporting the idea that substrate choice matters, not only total calories or potassium load [14,26,27]. The major characteristics of saccharolytic and proteolytic fermentation, including their dominant substrates, principal metabolites, and clinical relevance in CKD, are summarized in Table 1.

Among the best-studied gut-derived uremic toxins are indoxyl sulfate and p-cresyl sulfate, which arise from bacterial metabolism of tryptophan and tyrosine/phenylalanine, respectively [28,29]. Indole-3-acetic acid is another tryptophan-derived toxin [30]; trimethylamine N-oxide comes from microbial trimethylamine production followed by hepatic oxidation [29]; and phenyl-derived solutes such as phenyl sulfate or related phenyl compounds are increasingly recognized as part of the uremic metabolite spectrum. These compounds accumulate as kidney function declines and have been associated with oxidative stress, endothelial dysfunction, vascular stiffness, inflammation, renal fibrosis, cardiovascular events, and CKD progression in observational and experimental studies; however, the strength and causal relevance of these pathways differ among individual metabolites and evidence types [28,29,30]. The harmful roles of indoxyl sulfate and p-cresyl sulfate rest on observational human data together with mechanistic and preclinical studies; no randomized trial has yet demonstrated that lowering these toxins improves kidney or cardiovascular outcomes in CKD. Their pathogenic contribution should therefore be regarded as biologically plausible and associationally supported rather than clinically established.

The relationship between uremic toxins and potassium homeostasis is likely to be indirect and should not be overstated. Key factors that may attenuate the biological consequences of gut-derived uremic toxins include greater fermentable-fiber and resistant-starch availability, improved bowel transit, preservation of intestinal barrier integrity, and reduced proteolytic fermentation [25,31]. These features of minimally processed plant-food matrices may lower exposure to selected uremic metabolites while also supporting gastrointestinal potassium handling. However, there is currently insufficient evidence to conclude that lowering indoxyl sulfate, p-cresyl sulfate, or related uremic toxins directly lowers serum potassium or prevents hyperkalemia. The potential benefit therefore lies in addressing overlapping pathways (microbial metabolism, bowel function, inflammation, and potassium handling) rather than in a direct uremic toxin–potassium interaction. This proposed uremic toxin–potassium interaction is a hypothesis that has not been tested directly in humans and should be read as hypothesis-generating rather than as established clinical evidence.

Short-chain fatty acids provide the mechanistic counterpoint. Acetate, propionate, and butyrate help maintain epithelial barrier integrity and modulate mucosal and systemic immunity through G-protein-coupled receptor signaling and related pathways [22,32,33,34]. Experimental literature links SCFAs to sympathetic regulation, renin secretion, vascular tone, and blood pressure through receptors expressed in kidney tissue and blood vessels [33]. But translation to humans is not straightforward: a phase II prebiotic trial using acetylated/butyrylated resistant starch lowered ambulatory systolic blood pressure in untreated essential hypertension [35], whereas a 2024 randomized trial of oral butyrate in humans unexpectedly increased daytime systolic and diastolic pressure [36]. That contrast is exactly why CKD dietary recommendations should prioritize whole-food patterns and clinical monitoring rather than assume that any single SCFA intervention will replicate animal findings [33].At the level of evidence, the pathway linking SCFAs to inflammation, vascular tone, and blood pressure is supported chiefly by mechanistic and preclinical work and remains inconsistent in randomized human trials; it is presented here as biologically plausible rather than clinically established.

**Table 1:** Comparison of colonic fermentation patterns, dominant substrates, microbial metabolites, and their relevance to chronic kidney disease (CKD). The fermentation categories and their metabolites reflect established microbiological and mechanistic evidence, whereas their downstream clinical relevance in CKD is supported mainly by observational and preclinical data rather than randomized trials.

Dietary Pattern in the Colon	Dominant Substrate	Main Metabolites	Likely Relevance in CKD
Saccharolytic fermentation	Fiber, resistant starch, accessible carbohydrates	Acetate, propionate, butyrate	Better barrier support, immune regulation, faster transit, less substrate pressure for proteolytic toxin formation [26,37,38,39,40,41]
Proteolytic fermentation	Amino acids, especially aromatic amino acids	Indole derivatives, p-cresol derivatives, phenyl-derived toxins	Higher burden of protein-bound uremic toxins, oxidative stress, endothelial injury [42,43,44,45]
Mixed pattern with slow transit	Low fiber plus constipation	Lower SCFA efficiency and greater toxin retention	Common in CKD, may worsen inflammation and potassium handling [46,47,48,49]

## 4. How Fruits, Vegetables, and Legumes May Modulate the Axis

The most robust mechanistic rationale for plant foods in CKD is still dietary fiber. Fiber increases stool bulk, improves colonic transit, and provides fermentable substrate to the microbiota [27,50]. In CKD, meta-analytic evidence from randomized trials of fiber supplementation shows reductions in indoxyl sulfate and p-cresyl sulfate, although such trials are generally small and supplement-based rather than whole-food-based. Even so, they support the principle that restoring fermentable substrate can redirect microbial metabolism away from proteolytic toxin production [51]. Reductions in indoxyl sulfate and p-cresyl sulfate with fiber or resistant starch derive from small randomized trials and their meta-analyses and thus reach a clinical-evidence tier for the biomarker outcome; however, a corresponding effect on inflammation, CKD progression, or survival remains unproven, so these findings are best interpreted as surrogate-marker benefits rather than demonstrated clinical outcomes.

### 4.1. Resistant Starch

Resistant starch is especially relevant because it is abundant in foods such as legumes, under-ripe or green bananas, and some cooked-and-cooled starchy foods [52]. Resistant starch escapes small-intestinal digestion, reaches the colon, and is fermented to SCFAs, particularly butyrate [53]. Reviews and meta-analyses in CKD suggest resistant starch can reduce uremic toxins and may modestly improve renal markers, though inflammatory and hard clinical outcomes remain inconsistent [26,54]. In practice, legumes and culturally familiar starches prepared in ways that preserve some resistant starch may offer a food-based route to these benefits [26,55].

### 4.2. Polyphenols

Polyphenols add a second microbiota-facing mechanism. Fruits and vegetables provide flavonoids, anthocyanins, phenolic acids, and organosulfur compounds that interact bidirectionally with gut microbes: microbiota transform polyphenols into bioactive metabolites, while polyphenols can influence microbial composition and function [56]. CKD-focused reviews describe this as a plausible antioxidant and anti-inflammatory pathway, but the clinically meaningful evidence still lies more in food-pattern studies than in isolated nutraceutical supplementation [50]. That matters because the whole-food matrix also brings fiber, water, lower sodium density, and different absorption kinetics [57].

Polyphenols may also influence blood-pressure regulation through bidirectional interactions with the gut microbiota. Dietary polyphenols can modify microbial composition and function, while microbial transformation of polyphenols generates metabolites that may exert vascular and metabolic effects. These interactions may promote SCFA-producing microbial communities and contribute to improved intestinal barrier integrity, reduced inflammation, enhanced nitric oxide bioavailability, and modulation of pathways relevant to vascular tone and blood pressure. Polyphenol-rich plant foods may therefore contribute to the broader gut-kidney-blood pressure axis through combined microbial, antioxidant, anti-inflammatory, and vascular mechanisms. However, evidence remains heterogeneous and largely indirect, and a specific dose or polyphenol class cannot currently be identified as optimal for CKD. Importantly, direct evidence that dietary polyphenols alter intestinal potassium absorption is currently lacking; their relevance to potassium management should therefore be interpreted primarily through the whole-food matrix and microbiota-mediated pathways rather than as a demonstrated potassium-absorption mechanism [58,59]. The blood-pressure–related actions of polyphenols are supported predominantly by mechanistic and preclinical evidence with limited and heterogeneous clinical confirmation in CKD and should be regarded as proposed rather than established.

### 4.3. Plant Protein

Plant protein may also be part of the signal. Compared with animal-heavy diets, plant-predominant eating patterns usually deliver a lower dietary acid load and a more favorable fiber-to-protein ratio, which may reduce substrate availability for indolic and phenolic uremic toxins [46,60]. In an NHANES III analysis of adults with eGFR < 60 mL/min/1.73 m^2^, each 33% increase in the plant-protein-to-total-protein ratio was associated with a 23% lower hazard of all-cause mortality (HR 0.77, 95% CI 0.61–0.96), whereas no significant association was observed among participants with eGFR ≥ 60 mL/min/1.73 m^2^ (HR 0.88, 95% CI 0.74–1.04). However, this was an observational association and may reflect residual confounding because higher plant-protein intake often accompanies greater fiber intake, lower saturated-fat intake, and overall healthier dietary patterns [61].

Constipation represents an additional pathway through which plant-food intake may influence gut and potassium homeostasis in CKD. In CKD, constipation is associated with increased exposure to gut-derived uremic toxins and may also influence gastrointestinal potassium handling [62]. Fiber-rich foods can improve bowel regularity, and that may indirectly help both uremic toxin burden and potassium control. Dietary fiber may influence potassium handling primarily through gastrointestinal rather than direct transporter-mediated effects. Soluble and fermentable fibers increase stool water and bulk, while resistant starch is fermented to short-chain fatty acids; together, these effects can improve intestinal transit and may increase fecal potassium loss, particularly when colonic potassium secretion is an important adaptive pathway in advanced CKD. However, current evidence does not establish that dietary fiber consistently reduces the fractional intestinal absorption of potassium itself. The more defensible interpretation is that fiber modifies the gastrointestinal environment and potassium excretion rather than acting as a direct potassium-binding inhibitor. This mechanism may partly explain why indiscriminate potassium restriction can have unintended consequences. A low-fiber “renal” diet may worsen exactly the bowel dysfunction that contributes to toxin accumulation and potassium problems [47].

## 5. Potential Clinical Benefits in Hypertensive CKD

A potentially important benefit in hypertensive CKD is improved blood-pressure-related dietary physiology, although direct intervention evidence in CKD remains limited. Plant-predominant dietary patterns may contribute through lower sodium density, greater dietary alkali provision, and higher intake of fiber and other bioactive components. Observational CKD cohorts have associated greater adherence to DASH-like, Mediterranean, and plant-based dietary patterns with more favorable kidney and cardiovascular outcomes, but these associations do not establish that plant foods independently reduce blood pressure or slow CKD progression [63].

A second major benefit is metabolic acidosis correction. The most clinically persuasive intervention data come from fruits-and-vegetables alkali trials by Goraya and colleagues. Across CKD G3 and G4 studies, adding base-producing fruits and vegetables improved metabolic acidosis and reduced urinary kidney injury markers, often comparably to oral sodium bicarbonate; longer follow-up suggested preservation of eGFR and better cardiovascular risk markers in appropriately selected patients without baseline hyperkalemia. These trials do not prove that every potassium-rich plant food is safe for every CKD patient, but they strongly argue against equating “high potassium” with “universally harmful” [64]. Among the benefits discussed in this review, metabolic-acidosis correction with base-producing fruits and vegetables is supported by randomized clinical trials in selected CKD G3–G4 patients, albeit ones that excluded participants with baseline hyperkalemia.

Evidence for albuminuria and kidney-function decline is promising but still heterogeneous. KDIGO’s 2024 synthesis highlights cohort data from CRIC, NHANES, REGARDS, and other studies in which greater adherence to DASH-like patterns, higher plant-protein intake, or higher vegetable and nut intake was associated with lower CKD progression, lower kidney failure risk, or lower all-cause mortality. A randomized cardiovascular-prevention trial also found slower eGFR decline with a Mediterranean diet versus a low-fat diet in a CKD subgroup. Still, most evidence here is observational, and reverse causation is possible because high-risk patients may have already been restricting plant foods before study entry [63]. The kidney-outcome signal (slower eGFR decline and lower kidney-failure risk) is supported predominantly by observational cohort data, with a single randomized cardiovascular-prevention trial providing supportive but non-confirmatory subgroup evidence; whole-food plant-based interventions powered for hard kidney endpoints in CKD are still lacking.

Broader cardiovascular and metabolic benefits are biologically plausible and partially supported by the literature: better endothelial function, lower inflammation, improved insulin sensitivity, improved bowel function, better satiety, and improved dietary adequacy [27,50]. Patient-centered outcomes matter in CKD because overly restrictive diets are hard to sustain. Qualitative and review evidence indicates potassium restriction can erode food enjoyment, increase treatment burden, and reduce quality of life, whereas more flexible plant-inclusive approaches may improve variety and bowel comfort when clinically supervised [12]. The principal potential clinical benefits of fruits, vegetables, and legumes in hypertensive CKD, together with their proposed biological mechanisms and the current strength of supporting evidence, are summarized in Table 2. This evidence summary integrates KDIGO 2024, Goraya trials, fiber meta-analyses, and patient-experience literature.

## 6. Hyperkalemia Concern and Potassium Bioavailability

Hyperkalemia remains a real safety issue in CKD, but the physiology is more complex than “dietary potassium in equals serum potassium up.” Potassium homeostasis reflects renal excretion, adaptive gastrointestinal excretion, internal cellular distribution, and circadian and day-to-day variability [65,66,67]. As eGFR falls, the prevalence of hyperkalemia rises [68], particularly in patients with diabetes, albuminuria, or RAAS-inhibitor exposure [69,70,71,72]. CKD guidelines therefore treat hyperkalemia as a multifactorial problem rather than a purely dietary one [63]. The principal clinical and dietary determinants that influence serum potassium concentration and hyperkalemia risk in CKD are summarized in Table 3. This table summarizes KDIGO 2024 and major potassium-management reviews [63,73,74,75,76,77,78,79].

These determinants should be viewed as an interacting system rather than as independent predictors of serum potassium. Food matrix and processing determine the fraction of ingested potassium that becomes available for intestinal absorption; preparation and portion size modify the amount delivered to the intestine; fiber and bowel transit influence gastrointestinal handling and fecal potassium loss; and, after absorption, insulin, acid–base status, aldosterone activity, kidney function, and medications determine the distribution and elimination of potassium. Thus, the same nominal potassium intake can produce different serum potassium responses depending on the food form and the patient’s metabolic and renal context. That multifactorial view helps explain why the link between dietary potassium and serum potassium is often weak or inconsistent in nondialysis CKD [72,80]. KDIGO states that metabolic acidosis, hyperglycemia, constipation, and medications are often more important explanations for potassium abnormalities than diet. Observational studies using more detailed intake assessment or repeated 24 h urine collections likewise show weak associations between dietary potassium and serum potassium and, in some analyses, no clear association with hyperkalemia itself [63].

The clinical effect of potassium-rich plant foods also depends on their broader mineral composition. Sodium is the most clinically relevant counterpart because a lower sodium-to-potassium balance may favor natriuresis and blood-pressure control, although the blood-pressure response to higher potassium intake is less certain in CKD than in the general population. Magnesium may contribute to vascular and metabolic regulation and is frequently present alongside potassium in minimally processed plant foods, whereas calcium may influence vascular physiology but has no established direct role in determining potassium absorption. Phosphorus is particularly relevant to CKD because its dietary burden and bioavailability influence CKD-mineral and bone disorder, but phosphorus content should not be interpreted as a direct determinant of potassium bioavailability. Therefore, the clinical effect of a plant food should be considered as a composite mineral and food matrix profile rather than as potassium content alone [81].

Potassium content and potassium bioavailability should be distinguished conceptually. Total potassium reflects the amount present in a food, whereas bioavailability reflects the proportion released from the food matrix, available for intestinal uptake, and ultimately absorbed under specific gastrointestinal conditions. Consequently, foods with similar total potassium contents may not produce equivalent potassium exposure. The food matrix, degree of cellular disruption, fiber content, processing, preparation method, portion size, intestinal transit, and the patient’s gastrointestinal physiology can all modify potassium release and subsequent handling.

KDIGO 2024 explicitly advises limiting foods rich in bioavailable potassium, especially processed foods, for CKD G3–G5 patients with hyperkalemia risk. In the guideline’s synthesis, plant-based foods are estimated to have lower potassium absorption than animal foods or processed foods [63,82], and highly processed items rich in potassium additives, juices, and potassium-chloride salt substitutes may deliver more absorbable potassium than many fresh plant foods [82,83,84,85]. Supporting work on plant-food bioaccessibility also shows that potassium release from plant matrices varies substantially by food type and preparation [63]. The lower apparent absorption of potassium from many fresh plant foods may partly reflect the intact food matrix, in which potassium is distributed within cellular compartments and accompanied by fiber and other components that alter its release and intestinal exposure. In intact plant foods, potassium is distributed within cellular structures and embedded within a matrix containing fiber and other food components. Mechanical disruption, juicing, blending, drying, or industrial processing may alter this structure and increase potassium release into the gastrointestinal lumen. Dietary fiber does not appear to function as a direct potassium-binding agent that consistently prevents absorption; rather, its principal relevance is through modification of food structure, intestinal transit, stool characteristics, and potentially fecal potassium excretion. Thus, lower potassium bioavailability from some minimally processed plant foods should not be attributed to fiber alone but to the combined effects of the intact food matrix and gastrointestinal handling. By contrast, potassium added as soluble salts or concentrated in juices and processed foods is more readily dissociated and available for absorption. Animal foods may also provide a higher proportion of readily absorbable potassium, although the magnitude varies by food and preparation. KDIGO summarizes available evidence suggesting approximate potassium absorption of 50–60% from plant-based foods, compared with 70–90% from animal-based foods and approximately 90% from potassium salts used in processed foods; these values should be interpreted as indicative rather than universal [3,63,86].These absorption percentages are guideline-summarized estimates carrying wide uncertainty; direct measurements of fractional potassium absorption from specific whole plant foods in CKD are sparse, so the values illustrate relative differences between food forms rather than established clinical constants.

Most dietary potassium absorption occurs in the small intestine and is predominantly passive, driven by electrochemical and concentration gradients across the intestinal epithelium, with both paracellular and transcellular pathways contributing. The basolateral Na^+^/K^+^-ATPase maintains the intracellular electrochemical environment required for epithelial ion transport. After potassium is released from the food matrix, absorption occurs predominantly in the small intestine through largely passive paracellular and transcellular processes. However, the final effect of dietary potassium on serum potassium depends not only on intestinal absorption but also on intracellular potassium distribution, renal excretion, and adaptive gastrointestinal potassium secretion. These physiological processes may differ substantially according to kidney function, acid–base status, insulin activity, aldosterone signaling, bowel function, and medication use. Therefore, potassium bioavailability should be considered one component of overall potassium homeostasis rather than a direct predictor of serum potassium concentration. In the colon, potassium handling becomes particularly important in CKD because adaptive potassium secretion can increase as renal excretion declines, while active colonic potassium absorption can also occur under some conditions [87,88]. Thus, intestinal potassium bioavailability reflects both initial dietary release and subsequent epithelial transport, whereas serum potassium ultimately depends on intracellular distribution and renal and gastrointestinal excretion.

Gut microbiota may further influence potassium handling through effects on the colonic environment and potassium-secretory capacity. CKD-associated dysbiosis commonly includes depletion of butyrate-producing taxa such as Roseburia and Faecalibacterium prausnitzii, which may reduce short-chain fatty acid production and alter intestinal epithelial function. In the colon, potassium secretion is mediated in part by aldosterone-responsive large-conductance potassium (BK) channels; therefore, alterations in the gut environment, epithelial integrity, and neurohormonal signaling may modify the capacity for adaptive gastrointestinal potassium excretion when renal potassium elimination is impaired. This provides a plausible link between CKD-associated dysbiosis and potassium homeostasis, although the extent to which specific bacterial taxa directly regulate potassium absorption or secretion remains uncertain and has not been causally established in humans [89,90,91]. This microbiota–potassium link is therefore a proposed mechanism (hypothesis level), not established clinical or even confirmed mechanistic evidence in humans.

**Figure 2:**
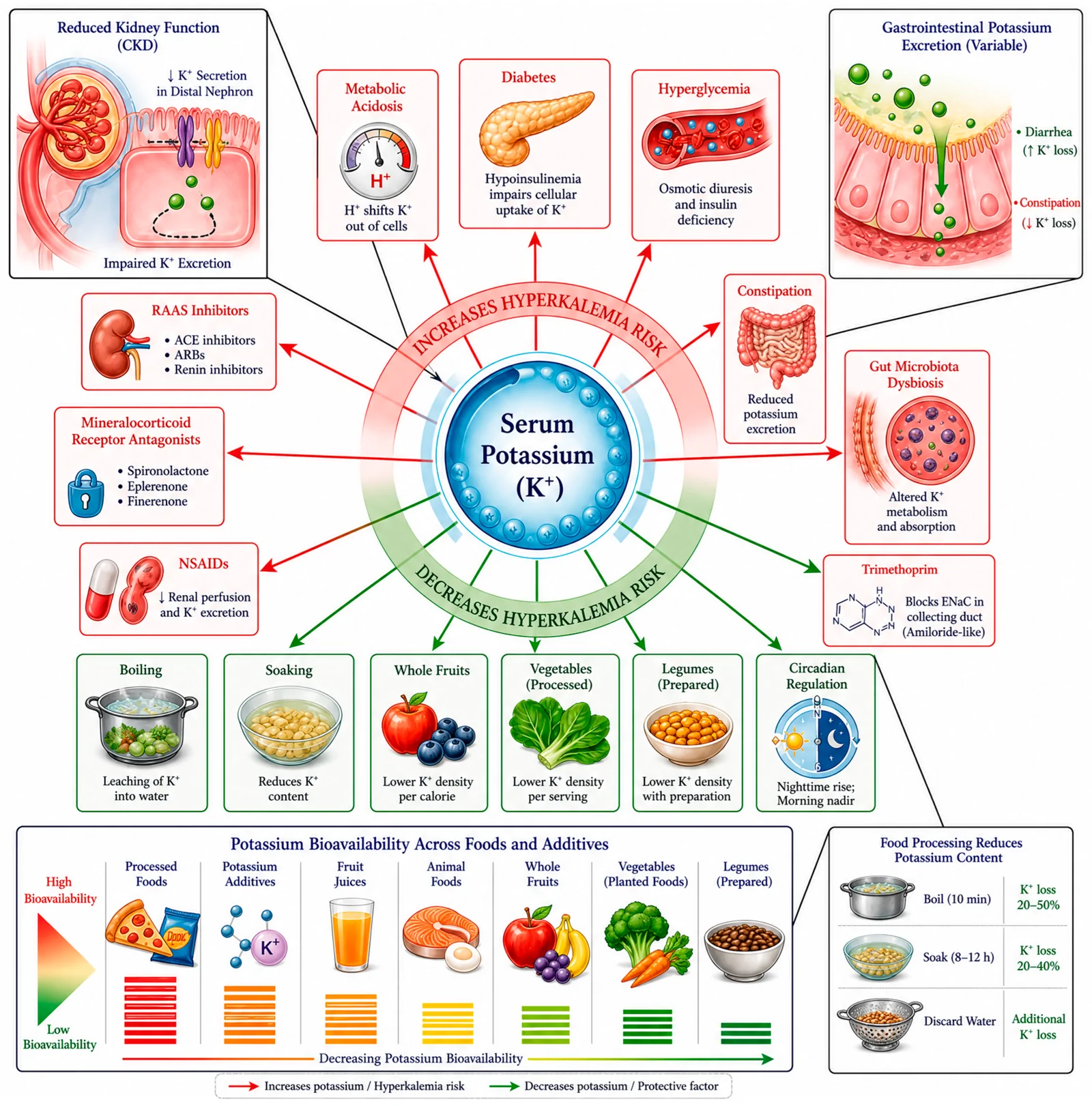
Potassium Is More Than Potassium: Determinants of Hyperkalemia Beyond Dietary Intake. Multiple determinants of serum potassium homeostasis in CKD. Hyperkalemia is influenced by kidney function, medications, metabolic disturbances, bowel function, and potassium bioavailability rather than dietary potassium content alone.

Preparation changes exposure further. Boiling, soaking, chopping, and discarding cooking water can materially reduce potassium in some vegetables and legumes, although the effect varies by food [92,93,94]. These methods primarily reduce potassium through leaching: potassium is present largely as a soluble ion in plant tissues and can diffuse into water when foods are cut, soaked, or heated. Boiling generally promotes greater transfer of potassium into cooking water because heat softens plant cell structures and increases contact between intracellular potassium and the surrounding water; discarding the cooking water therefore removes the leached potassium. As illustrated in Figure 2, boiling for approximately 10 min can reduce potassium content by about 20–50%, whereas soaking for 8–12 h can reduce potassium by approximately 20–40%, although the magnitude varies substantially by food type and preparation conditions. Discarding the soaking or cooking water provides additional removal of the potassium that has leached into the water. These effects are consistent with experimental evidence showing that potassium reduction varies according to food type, particle size, water-to-food ratio, temperature, and duration of treatment [92,93,94,95]. CKD-focused studies show that soaking and cooking dried and canned legumes can lower both potassium and phosphorus, potentially allowing more liberal inclusion in kidney diets [96]. Short hot-water soaking strategies may also reduce potassium while being more practical than lengthy leaching techniques [93]. The trade-off is that aggressive processing can also reduce palatability and some nutritional value, so the goal should be clinically targeted modification, not blanket demineralization of all plant foods [63]. Where quantitative estimates of potassium absorption are presented, these values should be interpreted as approximate ranges derived from guideline summaries and available experimental evidence rather than fixed values applicable to all foods or individuals. Potassium bioavailability varies substantially according to food matrix, preparation, gastrointestinal conditions, and study methodology; therefore, quantitative estimates should be used to illustrate relative differences between food forms rather than to predict an individual patient’s serum potassium response.

Nondietary risk modifiers often dominate in practice. KDIGO lists CKD stage, prior hyperkalemia, diabetes, hyperglycemia, metabolic acidosis, constipation, and medications such as ACE inhibitors, ARBs, spironolactone, eplerenone, finerenone, potassium-sparing diuretics, NSAIDs, and trimethoprim as key risk factors [63]. Importantly, KDIGO also emphasizes that hyperkalemia associated with RAAS inhibition should often be managed by potassium-lowering strategies rather than reflexive discontinuation of kidney- and cardio-protective therapy [63]. The multifactorial determinants of serum potassium homeostasis in CKD, including kidney function, medications, metabolic disturbances, bowel function, food processing, and potassium bioavailability, are integrated schematically in Figure 2.

## 7. Food-Group Evaluation, Risk Stratification, and Clinical Implementation

The food examples prioritized in this review were selected as practical exemplars rather than as an exhaustive or ranked list. Selection was driven by four considerations: their relevance to the gut-kidney axis through fiber, resistant starch, or other bioactive components; variation in potassium density and bioavailability; the availability of feasible preparation methods to modify potassium exposure; and their relevance to commonly consumed and culturally adaptable dietary patterns. Accordingly, whole fruits, vegetables, and minimally processed legumes were emphasized over isolated foods solely on the basis of their potassium content. For fruits, the most practical distinction is usually not “fruit or no fruit,” but whole fruit versus juice, dried fruit, or concentrates. Whole fruits preserve fiber and a slower eating pattern, whereas juices remove much of the fiber [97] and, in KDIGO’s framework, can provide relatively more absorbable potassium than fresh intact plant foods [10]. In CKD, the most defensible strategy is substitution and portion awareness, such as exchanging large volumes of juice or dried fruit for moderate portions of whole fruit, rather than complete elimination [63].

For vegetables, variety should be preserved whenever possible. Leafy vegetables, tomatoes, tubers, and cooked vegetable dishes differ meaningfully in potassium density, bioaccessibility, and preparation options [98]. Boiling can lower potassium in selected vegetables [99], while canned or preserved vegetables require attention to sodium and additives [100]. The clinical objective is to choose the right form and preparation method for the patient’s risk level, not to remove vegetables from the pattern altogether [63,94].

Legumes may be the most underused group in CKD nutrition. Beans, lentils, chickpeas, dry peas, and minimally processed soy foods provide plant protein together with dietary fiber and fermentable carbohydrates, including resistant starch, making them particularly relevant to dietary modulation of the gut-kidney axis [52,101]. From a practical perspective, microbiota modulation should focus on improving the intestinal environment rather than attempting to manipulate a single bacterial species. Increasing fermentable fiber and resistant starch through appropriately selected plant foods, maintaining regular bowel transit, avoiding unnecessary severe dietary restriction, and addressing constipation may support saccharolytic fermentation and gastrointestinal potassium handling. However, these approaches should currently be considered supportive strategies rather than established methods for directly lowering potassium absorption, and serum potassium should remain the clinical endpoint used to assess safety. They can also improve satiety and glycemic control [102,103] and reduce dependence on animal protein. Their potassium and phosphorus content requires respect, but CKD-specific food-processing studies show that soaking, boiling, draining, and portion control can make legumes more compatible with kidney diets than many handouts imply [93,96]. A related nuance is that highly processed plant-protein products may have more additives and a different mineral profile than whole legumes or simpler soy foods such as tofu and tempeh [96]. The principal characteristics of fruits, vegetables, and legumes, including their microbiota-related properties, potassium bioavailability, processing considerations, and practical dietary implications for hypertensive CKD, are compared in Table 4.

This logic also applies to regional and culturally relevant foods. In practice, clinicians may be discussing bananas or green bananas, beans and lentils, chickpeas, peas, soybeans, tofu, tempeh, leafy vegetables, tropical fruits, and cooked-and-cooled starchy foods. The clinically useful question is rarely whether these foods are universally “allowed” or “forbidden.” Their suitability depends on the patient’s potassium risk, bowel function, glycemic status, medication use, and preparation method. That culturally adaptive framing is more consistent with contemporary CKD guidance than rigid universal lists. A practical risk-stratified framework for implementing plant-food recommendations according to clinical profile and hyperkalemia risk is presented in Table 5.

A plant-rich dietary pattern should not be equated with unrestricted potassium intake in all patients with CKD. The potential nutritional and metabolic benefits of fruits, vegetables, and legumes must be balanced against the individual’s risk of hyperkalemia. This consideration is particularly important in patients with advanced CKD, reduced residual kidney function, established or recurrent hyperkalemia, recent acute kidney injury, metabolic acidosis, uncontrolled diabetes, or concurrent use of multiple potassium-raising medications. In these higher-risk settings, plant-food intake may still be possible, but food selection, portion size, preparation methods, and the intensity of biochemical monitoring should be individualized rather than universally liberalized or restricted.

**Figure 3:**
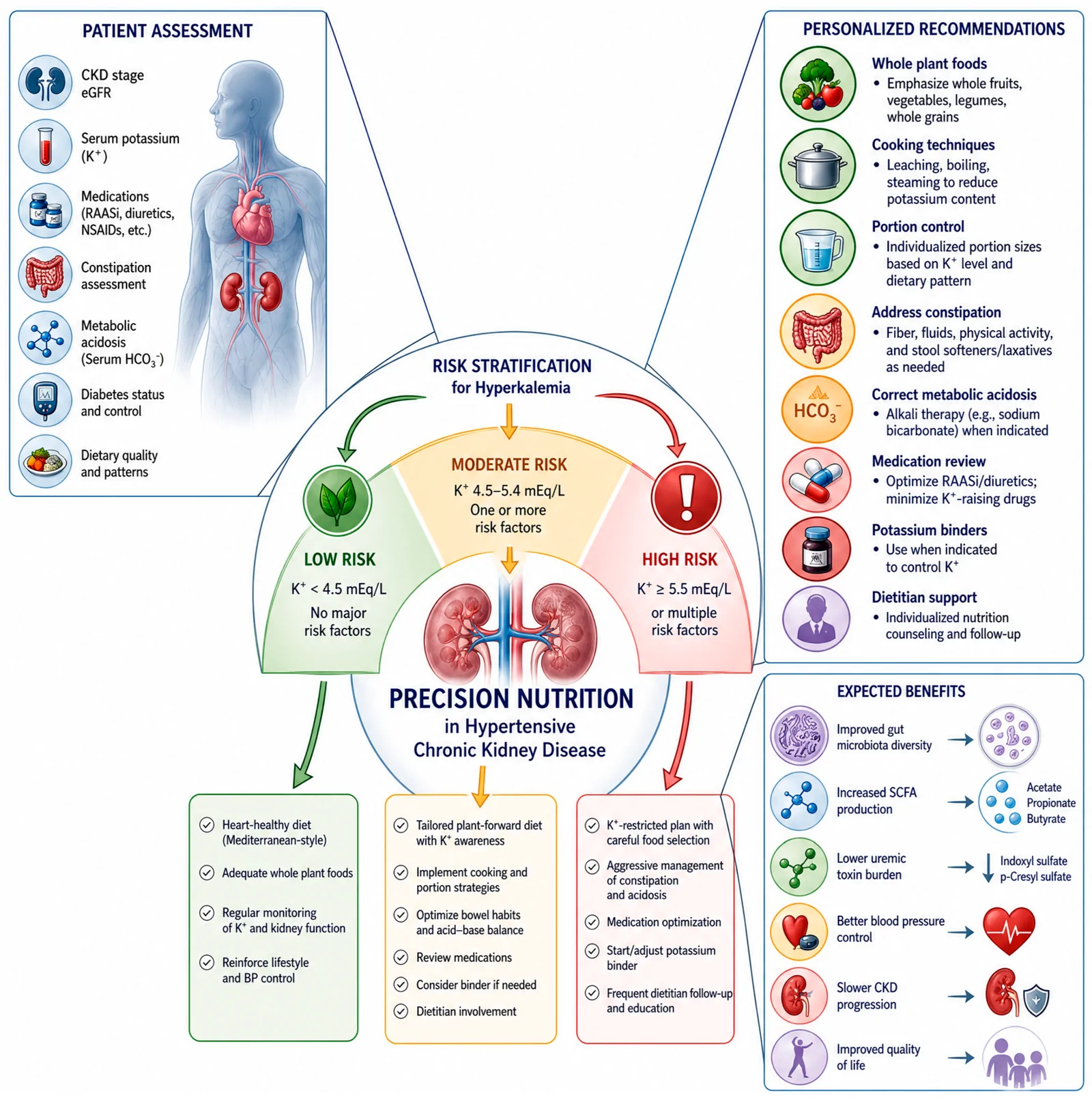
Precision Plant-Based Nutrition Framework for Hypertensive CKD. Proposed precision nutrition framework for integrating fruits, vegetables, and legumes into dietary management of hypertensive chronic kidney disease through individualized hyperkalemia risk assessment and gut-kidney axis optimization.

Monitoring should be guided by baseline risk and the magnitude of dietary change, not by a single universal interval. In most patients, the most important variables are serum potassium, bicarbonate, creatinine/eGFR, blood pressure, glucose control, bowel frequency, recent illness or AKI, and medication changes, especially initiation or dose escalation of RAAS inhibitors, MRAs, diuretics, or potassium binders. KDIGO specifically advises checking serum creatinine and potassium within 2–4 weeks of starting or changing a RAS inhibitor, with the exact timing depending on GFR and baseline potassium [63,86]. This framework synthesizes KDIGO 2024, potassium-management reviews, and practical CKD nutrition evidence [63,93,96,98,108]. The evidence discussed throughout this review can be translated into a practical precision nutrition framework that integrates patient assessment, hyperkalemia risk stratification, individualized dietary recommendations, and expected clinical outcomes, as illustrated in Figure 3.

Based on the evidence synthesized above, clinicians should not routinely exclude fruits, vegetables, and legumes solely because of their potassium content, but should instead assess overall hyperkalemia risk, including CKD stage, recent serum potassium, diabetes or hyperglycemia, metabolic acidosis, constipation, medications, and prior hyperkalemia. Whole and minimally processed plant foods should generally be prioritized over potassium-rich processed foods, juices, concentrates, potassium additives, and potassium-chloride salt substitutes. When potassium risk is elevated, portion size and preparation methods such as boiling, soaking, and draining can be used to reduce potassium exposure, although the magnitude of reduction varies by food and preparation method. Modifiable non-dietary contributors to hyperkalemia, including constipation, metabolic acidosis, poor glycemic control, and potassium-raising medications, should also be addressed rather than relying exclusively on dietary restriction. Subsequent dietary adjustments should be guided by monitoring of serum potassium, bicarbonate, creatinine/eGFR, blood pressure, bowel function, and relevant medication changes, with monitoring intensity tailored to baseline risk and the magnitude of dietary change. Patients with recurrent hyperkalemia, CKD G4–G5, AKI, uncontrolled diabetes or acidosis, or multiple potassium-raising medications may require closer monitoring, dietitian involvement, and, when clinically indicated, potassium-lowering therapy to facilitate a more flexible plant-food pattern.

Importantly, the inclusion of fruits, vegetables, and legumes should not be interpreted as universally safe for all patients with CKD. Hyperkalemia risk varies according to CKD stage and residual kidney function, baseline and previous serum potassium concentrations, acute changes in kidney function, diabetes and glycemic status, metabolic acidosis, bowel function, concurrent medications, and the magnitude and form of dietary potassium exposure. Therefore, plant-food recommendations should be individualized rather than applied as a uniform liberalization strategy. Patients with advanced CKD, recurrent hyperkalemia, recent acute kidney injury, or multiple potassium-raising risk factors may require more cautious food selection, portion adjustment, dietary modification, and closer biochemical monitoring.

Accordingly, a plant-rich dietary pattern should not be interpreted as unrestricted potassium intake. The clinical objective is not universal liberalization or blanket restriction, but individualized incorporation of plant foods according to potassium risk, kidney function, prior hyperkalemia, concurrent clinical factors, and ongoing biochemical monitoring.

## 8. Potassium-Lowering Therapies, Critical Appraisal, and Research Priorities

Newer potassium-lowering drugs are changing the dietary conversation [109,110,111]. KDIGO 2024 notes that patiromer and sodium zirconium cyclosilicate appear safer and better tolerated than older resins in long-term nonemergent hyperkalemia management, and it explicitly frames potassium-lowering therapy as a way to facilitate continuation of RAAS inhibition [63,112]. That same concept is now being extended to diet liberalization: feasibility work with sodium zirconium cyclosilicate in hyperkalemia-prone CKD reported improved dietary quality and greater healthy food intake with control of serum potassium, and ongoing trials are testing fruits-and-vegetables liberalization or plant-based diets supported by binders such as SZC or patiromer. The caveats are cost, access, pill burden, edema or gastrointestinal adverse effects, and the absence of definitive long-term outcome trials proving that binder-enabled plant-food liberalization improves kidney or patient-centered outcomes [63].

Adjunctive strategies remain essential. KDIGO’s stepwise approach to hyperkalemia emphasizes correcting metabolic acidosis, using diuretics appropriately, reviewing nonessential potassium-raising drugs, moderating dietary potassium with attention to source and processing, and addressing bowel dysfunction. This is clinically important because severe potassium restriction without correcting acidosis, constipation, or hyperglycemia may treat the laboratory number while leaving major drivers untouched [63].

The current evidence base has clear limitations. Dietary intervention studies are often small, short, and heterogeneous; definitions of “plant-based” vary substantially; food additives are rarely measured well; and many microbiome studies rely on 16S composition without functional metabolomics. Systematic reviews of potassium restriction also conclude that the evidence supporting low-potassium diets is of very low quality, with only a modest average reduction in serum potassium and uncertain effects on mortality or CKD progression [113]. Likewise, reductions in indoxyl sulfate or p-cresyl sulfate are encouraging but do not automatically translate into slower CKD progression or better survival [113].

Future research should therefore move beyond nutrient counting alone. The most important priorities are whole-food trials in clearly defined CKD stages and hyperkalemia-risk strata; direct comparisons of natural plant-food potassium with potassium additives and salt substitutes; measurement of true potassium bioavailability and urinary or fecal excretion; prespecified hyperkalemia safety outcomes; ambulatory blood-pressure assessment; simultaneous measurement of SCFAs, indoxyl sulfate, p-cresyl sulfate, TMAO, and related metabolites; functional microbiome methods such as shotgun metagenomics; careful recording of constipation, transit, acidosis, medication changes, and culturally relevant food preparation; and formal testing of whether potassium binders can safely support sustained plant-food intake in high-risk CKD. Ongoing protocol and feasibility trials show that the field is already moving in this direction [114].

As summarized in Table 6, the clinical case for individualized inclusion of plant foods rests on established evidence for acid–base benefit and on the weakness of the dietary-potassium–serum-potassium relationship, whereas the microbiota-, SCFA-, and uremic toxin-mediated mechanisms remain predominantly mechanistic, observational, or hypothesis-level and should not be presented as proven clinical effects.

## 9. Conclusions

The most clinically actionable conclusion is that fruits, vegetables, and legumes should not be restricted in CKD solely because their food composition tables list potassium. However, this should not be interpreted as implying that unrestricted consumption is safe for every patient with CKD. In hypertensive CKD, the suitability and amount of plant foods should be determined according to individual hyperkalemia risk, including CKD stage and kidney function, baseline and previous serum potassium concentrations, diabetes or hyperglycemia, metabolic acidosis, bowel function, concurrent medications, prior hyperkalemia, and the food matrix, preparation method, and portion size.

Within this individualized framework, fruits, vegetables, and legumes may contribute to better dietary quality, bowel function, and acid–base balance and may influence the gut-kidney axis through fiber, resistant starch, polyphenols, and related food matrix effects. However, many proposed microbiota-mediated mechanisms remain supported predominantly by mechanistic, experimental, and observational evidence, while large randomized clinical trials directly evaluating whole-food plant-based interventions and microbiome-mediated outcomes in CKD remain limited. Dietary liberalization, when appropriate, should therefore be accompanied by risk-based clinical and biochemical monitoring, with the intensity of monitoring tailored to the patient’s baseline risk and relevant changes in kidney function, medications, or dietary intake.

## Figures and Tables

**Table 2:** Potential clinical benefits of fruits, vegetables, and legumes in hypertensive chronic kidney disease (CKD): proposed mechanisms and current strength of evidence.

Potential Benefit	Most Plausible Mechanism	Current Strength of Evidence in CKD
Blood-pressure improvement	Lower sodium density, more alkali, favorable potassium–sodium balance, fiber/SCFA pathways	Moderate for dietary pattern associations; limited direct CKD RCT data (Observational or associational; limited direct CKD RCT evidence)
Better acid–base status	Reduced dietary acid load from fruits and vegetables	Relatively strong for selected CKD alkali trials (Established clinical randomized fruit-and-vegetable alkali trials, selected CKD G3–G4)
Lower uremic toxin burden	More fermentable substrate, less proteolytic fermentation	Moderate mechanistic support; modest trial evidence (Mechanistic or preclinical plus randomized biomarker (surrogate) evidence)
Better bowel function	Higher fiber and stool bulk, improved transit	Strong biologic plausibility and consistent clinical experience (Mechanistic plausibility and clinical experience; few controlled CKD trials)
Better diet quality and adherence	More variety, less reliance on ultra-processed foods	Moderate, but under-measured in formal trials (Observational)

**Table 3:** Major determinants of serum potassium and hyperkalemia risk in chronic kidney disease (CKD). These determinants are drawn from established clinical or guideline evidence (KDIGO 2024) and observational studies; the relative contribution of each determinant to an individual patient’s serum potassium has not been quantified in randomized dietary trials.

Determinant	Why It Changes Serum Potassium Risk
eGFR decline or AKI	Reduces renal potassium excretion
Diabetes, insulin deficiency, hyperglycemia	Impairs intracellular potassium shift and can raise extracellular potassium
Metabolic acidosis	Favors extracellular potassium redistribution and often coexists with CKD progression
Constipation and slow transit	Alters colonic potassium handling and may increase absorption/retention time
RAAS inhibitors, MRAs, potassium-sparing diuretics, NSAIDs, trimethoprim	Reduce renal potassium secretion or alter hormonal control
Food source and matrix	Changes bioavailability and postprandial exposure
Processing and additives	Potassium salts and concentrates can be more absorbable than intact foods

**Table 4:** Conceptual comparison of fruits, vegetables, and legumes according to microbiota-related properties, potassium bioavailability, and dietary implications in hypertensive chronic kidney disease (CKD).

Domain	Fruits	Vegetables	Legumes	Clinical Implication
Fermentable substrate	✓✓	✓✓✓	✓✓✓✓	Better gut microbiota [21,63]
SCFA production	Moderate	High	Relatively High	Improved blood pressure regulation [63,104]
Uremic toxin reduction	Moderate Potential	Higher Potential	Higher Potential	Potentially slower CKD progression [63,64,105]
Dietary acid load	Very Low	Very Low	Low	Better metabolic acidosis control [21,63,64,105]
Potassium bioavailability	Low	Moderate	Moderate	Depends on food matrix and preparation [63,93,98,106]
Effect of cooking	Small	Large	Large	Opportunity for potassium reduction [63,93,98]
Processing risk	Juice > whole fruit	Processed vegetables	Processed protein isolates	Prefer minimally processed foods [63,106]
Clinical recommendation	Encourage whole fruit	Encourage cooked vegetables	Encourage soaked/boiled legumes	Individualize according to CKD stage [63,93,107]

The qualitative descriptors (e.g., “Highest,” “High,” and “Moderate”) represent conceptual and relative estimates based primarily on the typical content and availability of fermentable dietary fiber and resistant starch, rather than results from direct head-to-head comparative trials among the three food groups. These ratings are intended to illustrate potential differences in microbiota-related effects and should not be interpreted as definitive rankings of clinical efficacy or uremic toxin reduction.

**Table 5:** Risk-stratified practical strategies for incorporating fruits, vegetables, and legumes in adults with hypertensive chronic kidney disease (CKD).

Typical Profile	Practical Plant-Food Strategy
Stable kidney function, normal serum potassium, no recent hyperkalemia, no major acidosis, good bowel function	Gradually increase minimally processed fruits, vegetables, and legumes; emphasize variety; routine lab follow-up
CKD G3b–G4, serum potassium near upper limit, diabetes, constipation, acidosis, RAASi or MRA use	Moderate portions, choose lower-bioavailable forms, use boiling/soaking where needed, avoid potassium additives and salt substitutes, monitor more closely after changes
Recurrent hyperkalemia, CKD G4–G5 or AKI, uncontrolled diabetes or acidosis, multiple potassium-raising drugs	Individualized modification rather than blanket exclusion; prioritize correction of non-dietary drivers; dietitian involvement; consider binder-supported liberalization when indicated

**Table 6:** Evidence-level classification of the principal claims of this review, distinguishing established clinical evidence from observational, mechanistic/preclinical, and hypothesis-level evidence.

Claim or Domain	Evidence Tier	Principal Basis	Key Limitation
Potassium homeostasis: dietary potassium weakly predicts serum potassium; control is multifactorial	Established clinical/guideline + observational	KDIGO 2024; CKD cohorts using repeated 24 h urine collections	Few CKD RCTs manipulate food-form potassium with serum-potassium endpoints
Potassium bioavailability: ~50–60% (plant) vs. ~70–90% (animal) vs. ~90% (additives/salt substitutes)	Guideline-summarized estimates	KDIGO synthesis; food bioaccessibility studies	Wide variation by matrix/preparation; sparse direct absorption data in CKD
Fiber/resistant starch lower indoxyl sulfate and p-cresyl sulfate	Randomized (surrogate biomarker)	Small RCTs and their meta-analyses	Surrogate markers; no proven effect on CKD progression or survival
Gut-derived uremic toxins contribute to inflammation, vascular injury, and CKD progression	Observational + mechanistic/preclinical	Human cohorts; cell and animal studies	No RCT shows that lowering toxins improves hard kidney or cardiovascular outcomes
Inflammation reduced via SCFAs and polyphenols along the gut-kidney axis	Mechanistic/preclinical	In vitro, animal, and translational human studies	Human anti-inflammatory benefit inconsistent; oral butyrate raised BP in one RCT
Gut microbiota/SCFAs modulate colonic potassium secretion	Proposed mechanism (hypothesis)	Preclinical and indirect physiological reasoning	Not causally established in humans
Metabolic-acidosis correction with base-producing fruits and vegetables	Established clinical (RCT)	Goraya CKD G3–G4 fruit-and-vegetable alkali trials	Selected patients; baseline hyperkalemia excluded
Kidney outcomes: slower eGFR decline and lower kidney-failure risk	Observational (+one RCT subgroup)	CRIC, NHANES, REGARDS cohorts; Mediterranean-diet CVD trial subgroup	Reverse causation possible; no whole-food RCT powered for kidney endpoints
Blood-pressure improvement with plant-predominant patterns	Observational patterns + limited RCT	DASH-like and Mediterranean cohorts	Direct CKD RCT data limited; potassium–BP response uncertain in CKD

## Data Availability

No new data were created or analyzed in this study. Data sharing does not apply to this article.
